# Mask-Induced Partial Transection of the External Ear Requiring Complex Surgical Reconstruction

**DOI:** 10.7759/cureus.25390

**Published:** 2022-05-27

**Authors:** Bharat Koti, Sahil Zaveri, Bhavin Shah, Shubhankar Anand, Ariana R Tagliaferri

**Affiliations:** 1 Surgery, Mount Sinai Hospital, Chicago, USA; 2 Internal Medicine, State University of New York (SUNY) Downstate Health Sciences University, Brooklyn, USA; 3 Family Medicine, Mercy Health - St. Rita's Medical Center, Lima, USA; 4 Internal Medicine, Garnet Health Medical Center, Middletown, USA; 5 Internal Medicine, St. Joseph Regional Medical Center, Paterson, USA

**Keywords:** covid-19, external ear, ear reconstruction, mask injury, pressure ulcer

## Abstract

During the COVID-19 pandemic, wearing masks to prevent the spread of infection has been imperative. Though many wear N-95 masks with circumferential head straps, the use of surgical ear loop-style masks has increased. Dermatologic complications, such as contact dermatitis, psoriasis, and local irritation, have been described in several reports. One such complication has been pressure injury to the external ear, secondary to friction from the ear loops. While external ear pressure ulcers caused by mask-wearing have already been observed, injuries extensive enough to require surgical reconstruction have yet to be described. Herein, we present a unique case of an elderly male with a severe external ear deformity caused by prolonged, uninterrupted mask-wearing that was treated with a complex ear reconstruction. The pressure caused a full-thickness erosion of the helical and conchal cartilage with partial auricle amputation from constant mask wear. We describe an unusual and interesting problem caused indirectly by the coronavirus pandemic and discuss potential methods to protect oneself against skin injury from mask usage while simultaneously preventing viral transmission.

## Introduction

Since the beginning of the coronavirus pandemic, the utilization of face masks has become the most widely practiced safety precaution besides hand hygiene and social distancing. Evidence shows that using face masks and physical distancing more than six feet apart can slow the transmission of SARS-CoV-2 [1]. While the CDC is gradually relaxing the recommendations for mask usage in public settings, face masks in areas with a high probability of viral transmissions, such as large crowds and transportation hubs, are still strongly recommended for individual protection [2].

Face masks and other personal protective equipment (PPE) are essential to protecting the public and healthcare workers alike, who have a high likelihood of coronavirus exposure. There have been many reports of dermatologic injuries, especially with incorrect or prolonged use. Skin breakdown, acne, and device-related pressure injuries have been shared among healthcare personnel required to wear protective face masks, goggles, and face shields [3,4]. Notably, face masks with elastic loops have been reported to cause pressure injuries to the ear, an area that is already susceptible to trauma, inflammation, and malignancy due to its anatomic location [5]. This risk is increased in the elderly population due to increased fragility. In one retrospective study, patients over 60 years of age had a relatively high incidence of dermatologic disease from various etiologies in the postauricular and antihelix areas of the ear [6].

Here, we present a unique case of an elderly patient from a long-term care facility who suffered a severe left pinna pressure deformity due to prolonged use of a facial mask with elastic loops. This manuscript highlights a unique complication of PPE usage, discusses the challenges plastic surgeons face with ear reconstructions, and explores potential methods for dermatologic protection during the use of facial masks.

## Case presentation

**Figure 1:**
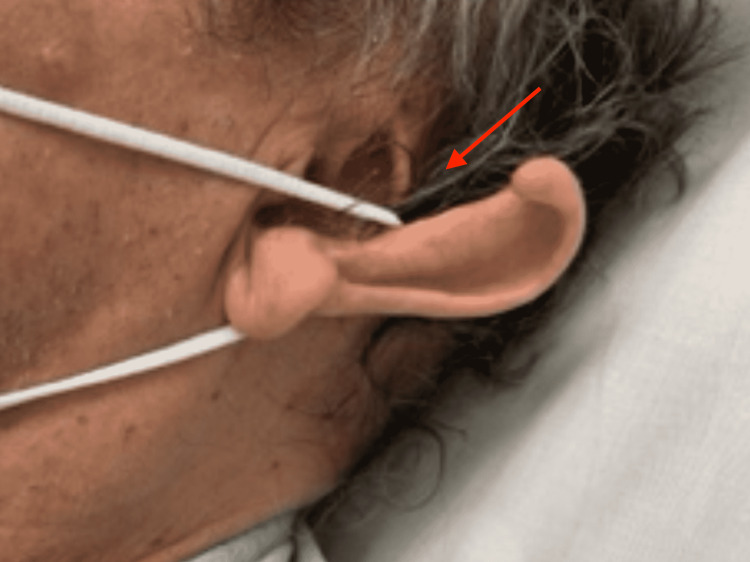
Preoperative external ear demonstrates partial transection. The red arrow indicates partial transection of the external ear from the helical rim to the concha, with completely healed skin edges.

A 65-year-old male with a history of disorganized schizophrenia presented to the otolaryngology clinic with a partial transection from the concha to his left helical rim from the persistent wearing of a disposable face mask with elastic ear loops (Figure 1). He was transported from his long-term care facility with a limited history due to the patient’s baseline mental status. There was no evidence of infection or ulceration along the separated edges of the ear. The skin edges were completely healed, extending over 50% of the length of the ear. Details of the procedure were discussed, and informed consent was obtained from the patient’s brother, his healthcare proxy.

**Figure 2:**
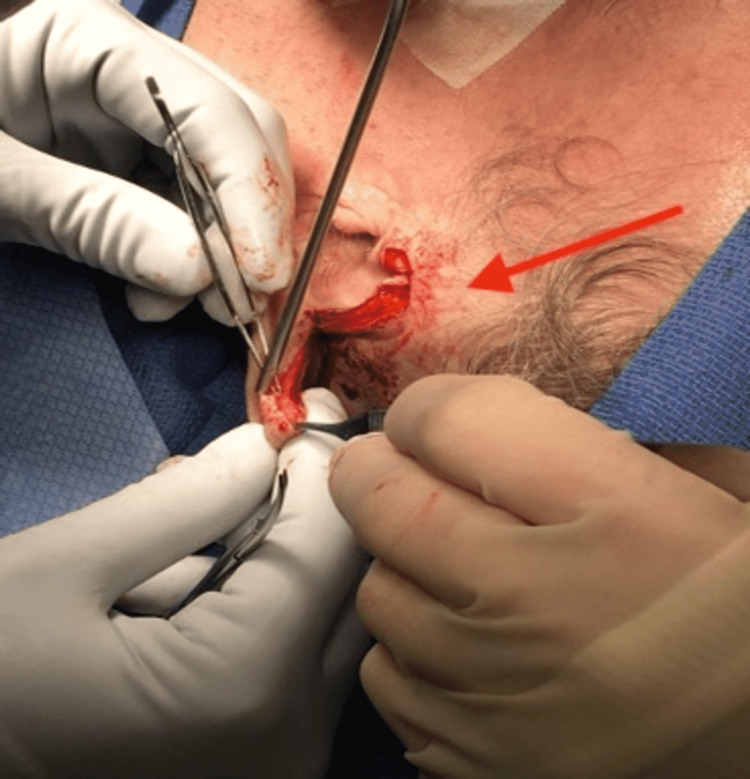
Intraoperative complex ear reconstruction. The red arrow demonstrates helical root realignment and approximation into the temporal fascia.

In preparation for the procedure, the patient was placed in a supine position and connected to cardiac monitoring. Prophylactic antibiotics were administered, and the sterile field was prepped. Intraoperatively, the edges of both ears were incised and dissected free (Figure 2). The soft tissue had intact cartilage on both sides with clear delineation. Once free, the posterior skin at the helix was re-approximated across the entire length of the ear. This was followed by a realignment of the cartilage, and then the helical root was repositioned into the temporal fascia. The posterior layer was approximated with a resorbable suture. The cartilage was then approximated with a Monocryl suture. Lastly, the anterior surface was approximated with a resorbable suture.

**Figure 3:**
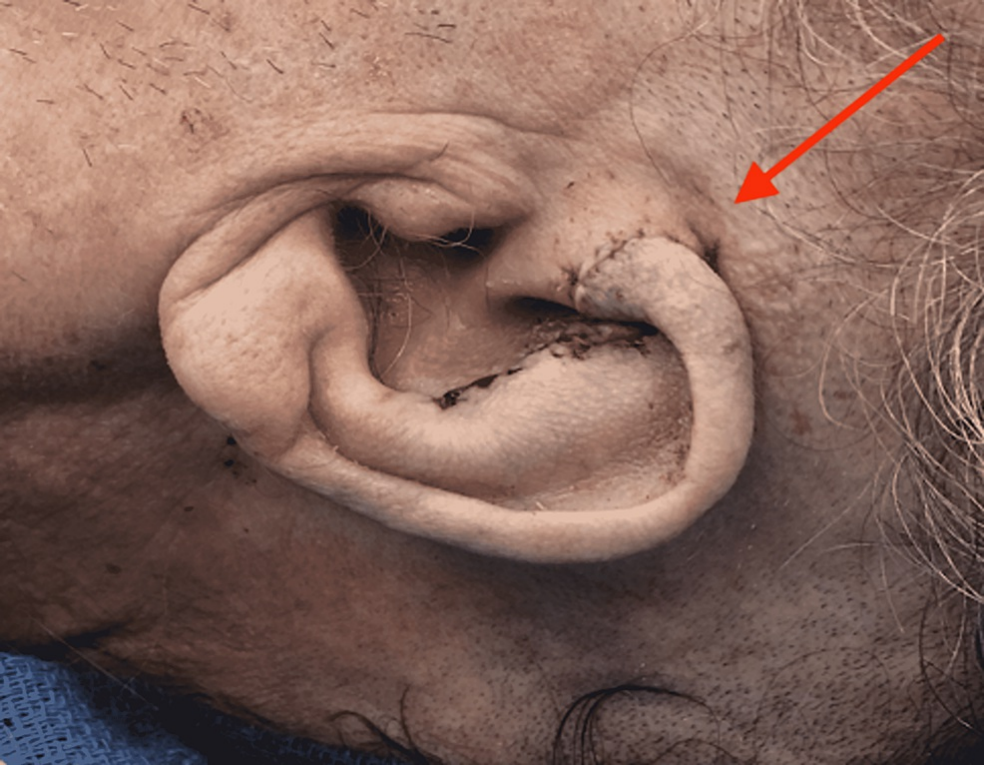
Postoperative follow-up reveals a well-healed ear. The red arrow demonstrates postoperative results from the complex ear reconstruction without evidence of infection.

After approximation of the skin layers, a sterile dressing was applied with a fluffed 4x4 gauze, both anterior and posterior to the ear, with a stockinette holding it in place (Figure 3). The patient was sent back to his long-term facility care after an unremarkable postoperative course with strict instructions for nursing to keep a face shield on the patient to avoid external pressure on the ear until his next clinic visit. At the follow-up visit two weeks later, the incision was healing well, without evidence of oozing, ulceration, drainage, or erythema. In addition, the patient denied any postoperative fever, chills, or myalgias.

## Discussion

Facial masks have been adopted as a global practice to protect oneself and others during the COVID-19 pandemic. However, various dermatologic complications can arise from prolonged or incorrect facial masks and PPE usage. Specifically, complications seen with disposable ear loop-style masks include contact dermatitis, psoriasis, and acute shear stress on the external ear, resulting in ulceration [5]. An example was a case report describing a Koebner phenomenon, a psoriatic lesion secondary to trauma found on the supra-auricular area [7]. While current literature describes mask-induced exacerbations of underlying chronic skin disorders as well as ulcerations of the external ear due to mask use, our case highlights a rare and severe external ear injury that required complex surgical reconstruction.

The specific anatomy of the ear presents a particularly distinctive challenge for the reconstructive surgeon. The structure of the external ear is supported by cartilage, and the skin that covers the pinna is covered in sebaceous glands, which protect the ear from desiccation and cracking. In addition, the external auditory canal is lined with glands that produce cerumen, which traps small foreign objects that enter through the external auditory meatus. To achieve a favorable outcome, appropriate anatomical knowledge of the innervation, blood supply, aesthetics, and careful attention to the contralateral ear are all required [8].

It is essential that various methods of skin protection while using PPE are explored. For example, broadening the ear loop with tape is suggested to increase the surface area in contact with the skin, thereby reducing the overall pressure. Another technique is to wrap the ear loop around spectacle handles in those who wear them [9]. In addition, the National Pressure Injury Advisory Panel described numerous methods to protect skin from mask-induced friction, including the use of barrier creams, moisturizers, skin sealants, and frequent PPE offloading [10]. In a recent study, a lubricant composed of 20% beeswax, 40% mineral oil, and 40% olive oil provided maximum protection against skin-PPE shear stress in study participants [11]. Furthermore, masks secured with straps behind the head should be considered as an alternative to ear loop-style masks as they allow the wearer to adjust straps to their level of comfort and reduce pressure on the skin. Together, these studies highlight several potential methods to protect one’s skin while simultaneously defending against SARS-CoV-2 infection.

## Conclusions

In the current coronavirus pandemic, PPE has become necessary to protect oneself and others from spreading COVID-19. However, it is also crucial to recognize the risk of skin injuries from prolonged and incorrect PPE use. The ear is especially prone to these complications given the pliability and fragility of the skin, location exposing it to traumatic injury, and as it simply serves as an anchor for loop-style face masks. While effective surgical management of the injured ear has been described in this manuscript, it is still essential to explore prophylactic methods to protect the ear during extensive PPE usage. Protection against coronavirus infection is still paramount during the pandemic, but it should not lead to injury in the process.
